# Diazepam-induced loss of inhibitory synapses mediated by PLCδ/ Ca^**2+**^/calcineurin signalling downstream of GABAA receptors

**DOI:** 10.1038/s41380-018-0100-y

**Published:** 2018-06-14

**Authors:** Martin W. Nicholson, Aaron Sweeney, Eva Pekle, Sabina Alam, Afia B. Ali, Michael Duchen, Jasmina N. Jovanovic

**Affiliations:** 1 UCL School of Pharmacy, University College London, London, WC1N 1AX UK; 2 Neuroscience, Physiology and Pharmacology, University College London, WC1E 6BT, London, UK

**Keywords:** Neuroscience, Psychiatric disorders

## Abstract

Benzodiazepines facilitate the inhibitory actions of GABA by binding to γ-aminobutyric acid type A receptors (GABA_A_Rs), GABA-gated chloride/bicarbonate channels, which are the key mediators of transmission at inhibitory synapses in the brain. This activity underpins potent anxiolytic, anticonvulsant and hypnotic effects of benzodiazepines in patients. However, extended benzodiazepine treatments lead to development of tolerance, a process which, despite its important therapeutic implications, remains poorly characterised. Here we report that prolonged exposure to diazepam, the most widely used benzodiazepine in clinic, leads to a gradual disruption of neuronal inhibitory GABAergic synapses. The loss of synapses and the preceding, time- and dose-dependent decrease in surface levels of GABA_A_Rs, mediated by dynamin-dependent internalisation, were blocked by Ro 15-1788, a competitive benzodiazepine antagonist, and bicuculline, a competitive GABA antagonist, indicating that prolonged enhancement of GABA_A_R activity by diazepam is integral to the underlying molecular mechanism. Characterisation of this mechanism has revealed a metabotropic-type signalling downstream of GABA_A_Rs, involving mobilisation of Ca^2+^ from the intracellular stores and activation of the Ca^2+^/calmodulin-dependent phosphatase calcineurin, which, in turn, dephosphorylates GABA_A_Rs and promotes their endocytosis, leading to disassembly of inhibitory synapses. Furthermore, functional coupling between GABA_A_Rs and Ca^2+^ stores was sensitive to phospholipase C (PLC) inhibition by U73122, and regulated by PLCδ, a PLC isoform found in direct association with GABA_A_Rs. Thus, a PLCδ/Ca^2+^/calcineurin signalling cascade converts the initial enhancement of GABA_A_Rs by benzodiazepines to a long-term downregulation of GABAergic synapses, this potentially underpinning the development of pharmacological and behavioural tolerance to these widely prescribed drugs.

## Introduction

Benzodiazepines are among the most widely prescribed class of drugs worldwide. Due to their rapid anxiolytic, sedative-hypnotic, anticonvulsant and muscle relaxant effects, they are prescribed for various conditions, most prominently anxiety, insomnia and epileptic seizures [1]. Among these disorders, anxiety, often coinciding with depression, is one of the most common mental health problems in the world (World Health Organization), and is the largest cause of sickness absence in the UK [2], with ~1 in 6 adults being chronically affected according to Mental Health Foundation UK. Although benzodiazepines are very effective initially, the major limitation to their long-term use is the development of tolerance to their pharmacological effects, as well as dependence, resulting in severe withdrawal symptoms upon drug cessation [1, 3]. Nevertheless, the estimated prevalence of long-term use among patients prescribed with benzodiazepine ranges from 25 to 76%, which is equivalent to 2–7.5% of the general population [4]. Although benzodiazepines have been prescribed for over 50 years, the molecular mechanisms leading to tolerance are still poorly understood.

In the nervous system, benzodiazepines bind exclusively to the GABA_A_Rs, the main inhibitory receptors in the brain, and allosterically enhance their responsiveness to GABA [5–7]. GABA_A_Rs are GABA-gated chloride/bicarbonate channels built up as hetero-pentamers from a pool of 16 different subunits classified as α(1–6), β(1–3), γ(1–3), δ, ε, π and θ, the combination of which determines physiological and pharmacological properties, as well as the tissue and subcellular distribution of these receptors. Benzodiazepines selectively bind to γ_2_ subunit-containing GABA_A_Rs, which are specifically localised to GABAergic inhibitory synapses, thereby enhancing the strength of synaptic inhibition in the brain. In addition to the γ_2_ subunit, synaptic GABA_A_Rs incorporate two α subunits (α_1_, α_2_, α_3_ or α_5_) and two β subunits (β_2_ or β_3_), with the benzodiazepine binding site residing at the interface between the γ_2_ subunit and one of the α subunits [8]. In neurons, synaptic GABA_A_Rs are clustered in the vicinity of presynaptic GABA-releasing terminals, to maximise the efficacy of transmission, but they are also mobile and dynamic, being continuously trafficked between the cytoplasm and the plasma membrane and, once at the cell surface, in and out of synapses [9].

Tolerance to specific benzodiazepines develops at differing rates and degrees in patients, with sedative and hypnotic tolerance within days, and anticonvulsant and anxiolytic tolerance within weeks, as these effects are mediated by specific subtypes of synaptic GABA_A_Rs in the brain [10, 11]. Diazepam, the most widely used anxiolytic drug in the clinic, is associated with prominent side effects such as sedation, ataxia and cognitive impairments, due to its non-selective binding to and modulation of all synaptic GABA_A_R subtypes [1]. Believed to be an adaptation to chronic enhancement of GABAergic signalling, tolerance has been correlated to the observed uncoupling between benzodiazepines and GABA binding sites [12, 13], modifications in GABA_A_R subunit expression [14, 15], or changes in the level and/or signalling of other neurotransmitters [11]. Endocytosis of GABA_A_Rs has also been implicated in long-term effects of benzodiazepines, however the signalling mechanisms that regulate this process, and the effects on inhibitory synapse structure and function, remain poorly characterised [11].

We demonstrate here that prolonged treatment of neurones with diazepam leads to disruption of neuronal inhibitory GABAergic synapses as a consequence of prominent time- and dose-dependent downregulation of surface GABA_A_Rs, mediated by dynamin-dependent internalisation. In this process, prolonged activity of GABA_A_Rs triggers a metabotropic signalling pathway which involves mobilisation of intracellular Ca^2+^ and calcineurin-dependent dephosphorylation and endocytosis of GABA_A_Rs. This, in turn, is dependent on the activity of phospholipase C (PLC), and mediated, at least in part, by diazepam-modulated direct binding of PLCδ to GABA_A_Rs. Thus, a metabotropic PLCδ/Ca^2+^/calcineurin signalling cascade, activated by diazepam downstream of GABA_A_Rs, leads to a long-term downregulation of GABA_A_Rs in synapses in a negative feedback fashion, a process likely to underpin the cellular correlates of pharmacological tolerance to these drugs. As such, it provides us with a new repertoire of therapeutic drug targets that may pave the way to improving the outcomes of the long-term clinical use of benzodiazepines.

## Materials and methods

### Cell culture

Sprague–Dawley rats (UCL-BSU) were housed and sacrificed according to UK Home Office guidelines, following project approval by the UCL Ethics Committee. Primary cultures of cerebrocortical neurones were prepared as described previously [16]. Briefly, cortical tissue was dissected from embryonic day 16-17 (E16-17) rats, dissociated by trituration in Ca^2+^ and Mg^2+^-free Hepes-buffered saline solution (HBSS), and plated at the required density in serum-free neurobasal medium, on either 0.1 mg/ml poly-d-lysine-coated dishes or 0.1 mg/ml poly-l-lysine-coated glass coverslips or glass bottom dishes. Cultures were incubated in a humidified 37 °C/5% CO_2_ incubator (Heracell, Heareus, Germany), for up to 14 days prior to experimentation.

Stable α1β2-HEK293 cell line was maintained under selection with G418 disulphate (Neomycin; G5013-Sigma-Aldrich) and Zeocin (R25001-Invitrogen) [17], while a stable α1β2γ2-HEK293 cell line (Sanofi-Synthelabo, Paris, France) was maintained under selection with G418 disulphate [18].

### Immunocytochemistry and synaptic cluster analysis

Cortical neurones (32,500/cm^2^ plated on glass coverslips) were subjected to various treatments and processed for immunolabelling using β_2/3_- and γ_2_-specific antibodies to label the cell surface GABA_A_Rs, and, following permeabilization, VGAT and MAP2-specific antibodies, to label GABAergic terminals and dendrites, respectively. Following incubation with the corresponding Alexa-Fluor secondary antibodies (Supplementary Table S1), samples were imaged using a laser scanning confocal microscope (Zeiss LSM 710 Meta, Zeiss, Germany) with a ×63 oil-immersion objective.

The size and number of GABA_A_R clusters, and their colocalization with VGAT-terminals, were analysed using Zen2.1 software, as previously described [19]. As the imaging was done in separate channels, a threshold for each fluorophore was determined by the formula (Threshold = mean intensity + (2 × standard deviation)). GABA_A_R clusters were defined as immuno-reactivity greater than 0.1 μm^2^, with a mean fluorescence value greater than 2 × standard deviation of background fluorescence, which were present along the first 20 µm length of MAP2-positive primary dendrites. Colocalisation in separate channels (at least 50% overlap) was determined by overlaying the images. Synaptic elements size and numbers were analysed using Origin Pro 9.1 software, and the values were expressed as outlined in the figure legends. Normality tests were performed using the Shapiro–Wilk and Kolmogorov–Smirnov tests. After normality tests were performed on each of the groups, non-parametric statistical analysis was done using Mann–Whitney test with the confidence interval of 95%, as the groups showed non-Gaussian distribution.

### GABA_A_R internalisation assay

Cell-surface receptors were labelled in living cultured neurons or in α1β2-HEK293 cells transiently transfected with the myc-γ2 cDNA, as described previously [19]. Briefly, coverslips were incubated with ice-cold Buffer A (mM: 150 NaCl; 3 KCl; 2 MgCl_2_; 10 HEPES, pH 7.4; 5 Glucose), containing 0.35 M sucrose for 5 min, followed by incubation with mouse anti-β_2/3_ antibody (MAB341-MerckMillipore) for 30 min at 4 °C in Buffer A containing 0.35 M sucrose, 1 mM EGTA and 1% BSA. Cells were further incubated at 37 °C in Buffer A containing 1 mM CaCl_2_ and 5 µg/ml leupeptin, in the absence or presence of diazepam (1 µM, Tocris), for 1 h to allow internalisation of the labelled β_2/3_ subunit-containing GABA_A_Rs. Cells were fixed with 4% paraformaldehyde/4% sucrose/PBS (PFA/PBS) and processed for immunolabelling with anti-mouse Alexa-555 antibodies overnight at 4 °C. Cells were subsequently permeabilized and labelled with anti-mouse Alexa-488 antibodies (Supplementary Table S1) for 1 h at room temperature. Samples were imaged using a Zeiss LSM 710 confocal microscope as above.

### Cell surface ELISA

Cortical neurons (14 DIV, 100,000 cells/cm^2^ in 24-well plates) or α1β2-HEK293 cells (111,000 cells/cm^2^ in 24-well plates) transiently transfected with the myc-γ2 cDNA using nucleofection (Lonza, Switzerland), were subjected to treatments with vehicle (DMSO) or diazepam (1 µM), in the absence or presence of various reagents (Supplementary Table S2). All the treatments were done in duplicate. Cells were fixed with PFA/PBS, and changes in surface and total levels of GABA_A_Rs were detected using cell surface ELISA with β_2/3_ (1 µg/ml)- or myc (1 µg/ml)-specific mouse monoclonal primary antibody and HRP-conjugated anti-mouse secondary antibody [16]. Values were expressed as mean percentage of vehicle treated control (set at 100%) ± s.e.m., with the number of independent repeats of each experiment (*n*) indicated in the figure legends. Statistical analysis was carried out using ANOVA with Dunnett post-hoc analysis and data plotted using OriginPro 9.1.

### Electrophysiology

Whole-cell recordings were made from cortical neurons (14 DIV), which were treated with DMSO or diazepam for 72 h, as described before above. Patch pipettes (resistance 8–10 MΩ) were pulled from borosilicate glass tubing and filled with an internal solution containing (mM): 144 K-gluconate, 3 MgCl_2_, 0.2 EGTA, 2 Na_2_-ATP, 0.2 Na_2_-GTP, 10 HEPES pH 7.2–7.4, 300 mOsm. Spontaneous activity of the neurons was recorded in current clamp mode (SEC 05 L/H, NPI electronics, Tamm, Germany), in the presence of TTX (1 μM), D-AP5 (50 μM) and CNQX (20 μM, all from Tocris). Synaptic potentials recorded were amplified, low-pass filtered at 2 kHz, and digitised at 5 kHz using a CED 1401 interface and data acquisition programme, Signal 4.04 (Cambridge Electronic Design, Cambridge, UK), and analysed offline using Signal. Single sweep amplitudes were measured from the baseline to the peak of the IPSP, and selected for analysis if greater than 0.05 mV [19]. Statistical analysis was conducted using the Student’s t test and data plotted using OriginPro 9.1.

### Biochemical assays

GST pull-down assays were performed, as described previously [16] using either cortical cell lysates, GFP-PLCδ- or GFP-PRIP-transfected HEK293 cell lysates, or in vitro translated PLCδ (TNT Quick Coupled Transcription/Translation kit, Promega). For coimmunoprecipitation analysis, primary cortical neurons or α1β2γ2-HEK293 cells transfected with GFP-PLCδ, GFP-PRIP1 or both cDNAs using Calcium–phosphate procedure [20], were incubated in the absence or presence of diazepam (1 µM), or in the absence or presence of diazepam/isoguvacine, respectively, for 2 h, and lysed under nondenaturing conditions. Cell lysates were incubated with 10 μg of either nonspecific goat IgG, or GABA_A_R α1-specific antibody [21], followed by incubation with Protein G-Sepharose. Precipitated proteins were resolved using SDS–PAGE and immunoblotting conducted as described previously [16], using antibodies described in Supplementary Table S3. Protein concentration was determined using either Bradford or BCA assays (ThermoFisher).

### Intracellular Ca^2+^ imaging

Cortical neurones (14 DIV; 20,000 cells/cm^2^ in 35 mm glass bottom dishes) were incubated with 5 µM Fluo-4 AM/0.0025% pluronic F-127 (ThermoFisher) for 30 min at 37 °C in the Recording Buffer (in mM: 10 HEPES pH 7.35, 156 NaCl, 3 KCl, 1.25 KH_2_PO4, 10 d-glucose, 2 CaCl_2_) containing CNQX (20 µM), TTX (0.5 µM) and D-AP5 (50 µM) [22]. Intracellular Ca^2+^ was recorded using Zeiss LSM 880 confocal microscope at 37 °C using a ×40 oil-immersion objective and recordings were analysed using Zen2.1 SP2 software (Zeiss, Germany). To quantitate changes in fluorescence intensity, the ‘Region of Interest’ tool was used to monitor regions in the somas and dendrites. The maximal fluorescence values following diazepam application were normalised to the average baseline control before the addition of diazepam (F_t_/F_0_), and analysed using ANOVA followed by Bonferonni post hoc analysis in OriginPro 9.1 software.

### Fluorescent imaging of GFP-PH_PLCδ_ and DsRed-PRIP1

α1β2γ2-HEK293 cells (20,000 cells/cm^2^ in 35 mm glass bottom dish) were transfected with 0.2 μg GFP-PH_PLCδ_ [23] in the absence or presence of 0.2 μg DsRed-PRIP1 [24] cDNA using Effectene (Qiagen) and cultured for 24 h. The cells were then incubated with Calcein Blue AM/0.0025% pluronic F-127 (ThermoFisher) for 30 min at 37 °C in HBS (in mM 10 HEPES pH7.4, 150 NaCl, 3 KCl, 2 MgCl_2_, 5  D-glucose, 2  CaCl_2_). Calcein Blue, GFP-PH_PLCδ_, or DsRed-PRIP1 emission was collected at 360–450 nm, 500–560 nm or 570–600 nm, respectively. Images (12-bit resolution) were acquired every 5 s before and after the bath application of diazepam (1 μM) and isoguvacine (5 μM) for total of 15 min using Zeiss LSM 880 confocal microscope at 37 °C using the ×40 oil-immersion objective and analysed using Zen2.1 SP2 software (Zeiss, Germany). Alternatively, cells were treated for 1 h with diazepam (1 μM) and isoguvacine (5 μM) in HBS (in mM: 10 HEPES pH7.4, 150 NaCl, 3 KCl, 2 MgCl_2_, 5 D-glucose, 2  CaCl_2_) and subsequently fixed with 4% PFA/4% sucrose (w/v). Cell surface GABA_A_Rs were labelled with an anti-β2/3 antibody (BD17) and an Alexa Fluor 555 antibody. GFP-PH_PLCδ_ emission was collected at 500–560 nm and Alexa Fluor 555 emission was collected at 570–600 nm. To quantify changes in fluorescence, the ‘Profile’ tool was used to produce fluorescence profiles for each fluorophore. The fluorescence at the membrane (F_m_) and the fluorescence in the cytoplasm (F_c_), measured before and after bath application of diazepam/isoguvacine, were used to produce a fluorescence ratio F_m_/F_c_ which was analysed using Students *t*-test in OriginPro9.1 software.

### Statistical analysis

Statistical analyses were performed using OriginPro9.1 software. Each dataset was tested for normality using the Shapiro–Wilk test. For non-parametric data sets, the Kruskal–Walis test followed by Mann–Whitney test was used and the results were presented using boxplots showing the median, interquartile range, standard deviation and the mean, as specified in the figure legends. For parametric data sets, either the two-tailed Student’s *t*-test or ANOVA followed by Bonferonni post hoc analysis was used and the results were expressed as the mean ± standard error of the mean. The exact sample size and the number of independent experiments performed, description of the samples and statistical analyses done were also specified in the figure legends. The number of timed-pregnant rats/litters was minimised by utilising the same tissue in multiple experiments, including biochemical, immunocytochemical, electrophysiological and live cell imaging experiments. Values were considered to be statistically significant for *p* < 0.05 (*).

## Results

### Diazepam causes a time-dependent disassembly of GABAergic synapses

**Fig. 1:**
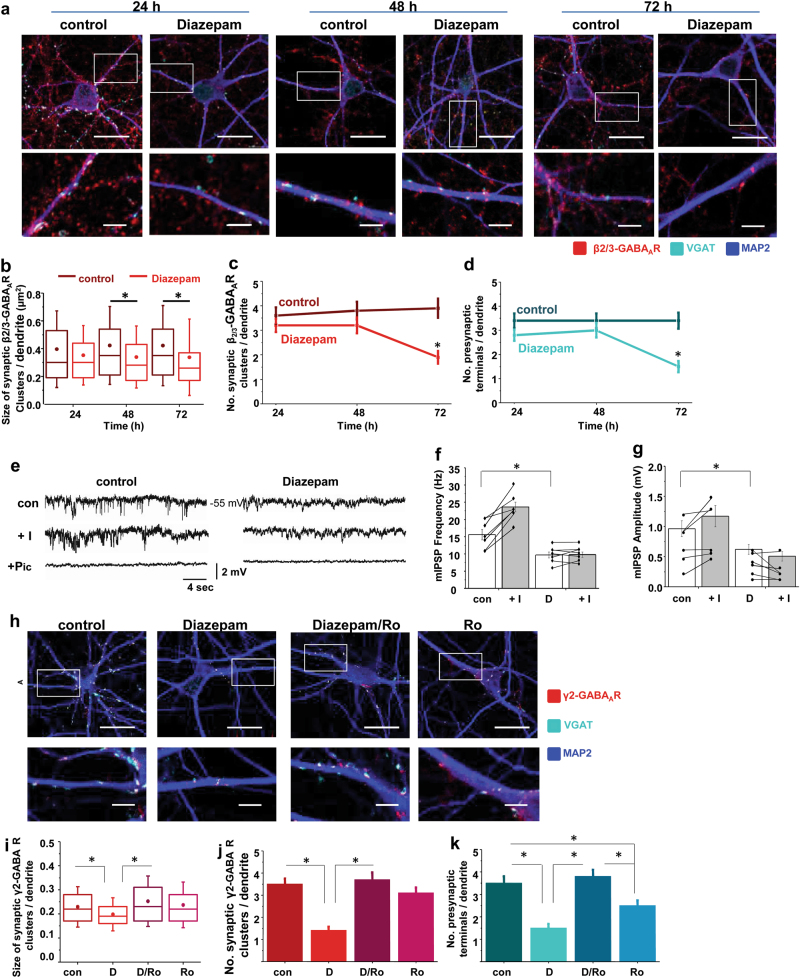
Diazepam causes a time-dependent breakdown of GABAergic inhibitory synapses upon direct binding to GABA_A_Rs. **a** Immunolabeling of postsynaptic GABA_A_R β_2/3_-containing clusters (red) and VGAT-positive presynaptic GABAergic terminals (cyan) along MAP2-positive primary dendrites (20 µm; blue) of cortical neurons in the absence or presence of diazepam (D; 1 μM), and the corresponding graphs showing a decrease over time in **b** size (median/line-IQRs; mean/dot ± s.d. whiskers; Mann–Whitney test, *p *<* *0.05*) and **c** number (mean ± s.e.m.; ANOVA/Bonferonni post-hoc test; *p *<* *0.05*) of synaptic β_2/3_ clusters, and **d** number of GABAergic terminals (mean ± s.e.m.; ANOVA/Bonferonni post-hoc test; *p *<* *0.05*) contacting *n* *=* *59*, *n* *=* *70*, *n* *=* *53* control (DMSO)-treated primary dendrites and *n* = 70, *n* = 67, *n* = 44 diazepam-treated primary dendrites for 24 h, 48 h, and 72 h, respectively. Total of *n* = 17, *n* = 18 and *n* = 15 control and *n* = 17, *n* = 17 and *n* = 17 diazepam-treated neurons, respectively, collected from two independent experiments, were analysed in each group. **e** Representative traces of mIPSPs recorded in cortical neurons after 72 h treatment with control (DMSO) or diazepam (D; 1 μM), before and after application of isoguvacine ( + I, 50 μM), followed by picrotoxin ( + Pic, 50 μM; scale refers to all conditions), and corresponding bar graphs (mean ± s.d.; Student’s *t*-test: *p < *0.05*) showing a diazepam-dependent decrease in **f** frequency and **g** amplitude of mIPSPs and the effects of isoguvacine ( + I, 50 μM; grey bars) from *n* = 7 control & *n* = 7 diazepam-treated cells collected from *n* = 3 independent experiments. **h** Immunolabelling of γ2-containing clusters (red) and VGAT-GABArgic terminals (cyan) along MAP2-positive dendrites (20 µm; blue) following 72 h treatment with control (DMSO), or diazepam (D; 1 μM), in the absence or presence of Ro 15-1788 (Ro; 25 μM), and the corresponding graphs showing a decrease in **i** size (median/line-IQRs; mean/dot ± s.d. whiskers; Mann–Whitney test, *p < 0.05), and **j** number of synaptic γ_2_ clusters (mean ± s.e.m.; ANOVA/Bonferonni post-hoc test*;* *p < 0.05), and **k** decrease in the number of GABAergic terminals (mean ± s.e.m.; ANOVA/Bonferonni post-hoc test; *p *<* 0.05) contacting *n* = 49 control-treated & *n* = 45, *n* = 53, *n* = 48 diazepam-, diazepam/Ro- or Ro-treated dendrites for 72 h, respectively. The total of *n* = 15, *n* = 14, *n* *=* 13, *n* = 16 neurons, respectively, collected from two independent experiments, were analysed in each group. Scale bars = 20 μm (**a**, **h**-upper row) and = 5 μm (**a**, **h**-lower row)

Binding of diazepam to a specific allosteric site harboured by the GABA_A_R at the interface between α and γ subunits leads to a rapid increase in channel gating [25, 26] and results in cumulative enhancement of GABA-mediated transmission at inhibitory synapses. However, under the conditions of sustained stimulation of GABA_A_Rs by diazepam (1 μM; concentration analogous to measured plasma concentration of diazepam taken orally by patients [27]) over the time course of 72 h, inhibitory GABAergic synapses underwent a gradual decline in structural integrity due to a significant reduction in the size (Fig. 1a, b; *p < 0.05) and number of dendritic postsynaptic GABA_A_R clusters (Fig. 1a, c; *p < 0.05), as well as the number of colocalised GABAergic presynaptic terminals (Fig. 1a, d; *p < 0.05), as observed in triple immunolabelling experiments with GABA_A_R-β_2/3_-, vesicular GABA transporter (VGAT)- and microtubule-associated protein (MAP2)-specific antibodies and confocal imaging in primary cerebrocortical neurones. The extrasynaptic GABA_A_R clusters monitored at the same time, which were significantly smaller in size, but larger in number than synaptic clusters, remained unaffected by diazepam (Supplementary Figure 1a and b). The total number of immunolabelled GABAergic or glutamatergic neurones remained unchanged (data not shown). Consistent with the observed structural changes in synapses was a prominent reduction in the frequency and amplitude (Fig. 1e–g; *p < 0.05) of miniature inhibitory postsynaptic potentials (mIPSPs), recorded in whole-cell current clamp mode, in neurones treated with diazepam for 72 h. When Ro 15-1788 (flumazenil), a specific competitive antagonist at benzodiazepine binding site on the GABA_A_R, was applied together with diazepam, the decrease in size (Fig. 1h, i; *p < 0.05) and number (Fig. 1h, j; *p < 0.05) of postsynaptic γ_2_-GABA_A_R clusters and the decrease in the number of colocalised GABAergic terminals (Fig. 1h, k; *p < 0.05) were completely abolished, indicating that direct diazepam binding to GABA_A_Rs was necessary for the observed loss of GABAergic synapses. However, Ro 15-1788 alone caused a decrease in the number of GABAergic terminals colocalised with the postsynaptic GABA_A_Rs, suggesting that it may have additional presynaptic effects that remain to be elucidated.

### Diazepam triggers internalisation of GABA_A_R receptors by activating calcineurin

**Fig. 2:**
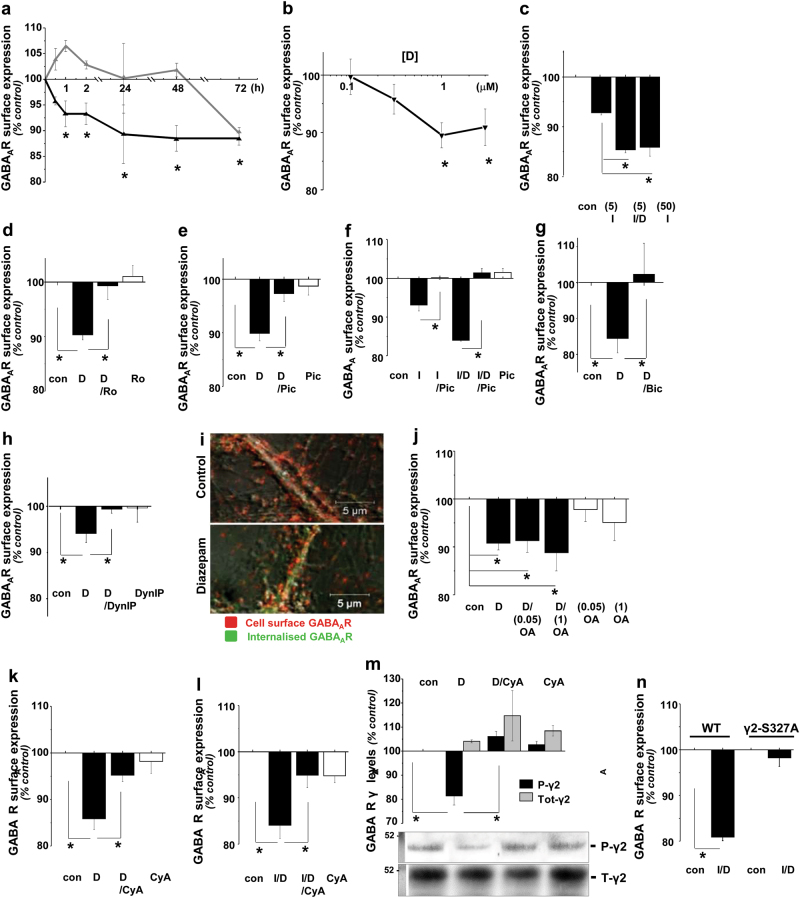
Time and dose-dependent internalisation of GABA_A_Rs in response to diazepam requires calcineurin activity. **a** Diazepam (D; 1 μM)-dependent decrease in surface (black) and total (grey) levels of GABA_A_Rs over time (*n* *=* *5*), and **b** in surface levels only in the presence of increasing doses for 2 h (*n* *=* *4)* in cortical neurones. **c** Decrease in surface GABA_A_Rs in response to low doses of isoguvacine (I; 5 μM) is potentiated by diazepam (D; 1 μM) in α_1_/β_2_/γ_2_^myc^-HEK293 cell line (*n* *=* *6*). **d**–**g** Diazepam (D; 1 μM)-dependent decrease in surface GABA_A_Rs is inhibited by Ro 15-1788 (Ro; 25 μM; **d**; *n* *=* *7*), picrotoxin (Pic; 50 μM; **e**; *n* *=* *5*), or bicuculline (Bic; 50 μM; **g**; *n* *=* *4*) in cortical neurones, and **f** picrotoxin (Pic; 50 μM; *n* *=* *6)* in α_1_/β_2_/γ_2_^myc^-HEK293 cell line. **h** Diazepam (D; 1 μM)-dependent decrease in surface GABA_A_Rs is inhibited by dynamin-inhibitory peptide (DynIP; 25 μM; *n* *=* *5*) in cortical neurones following 2 h treatments. **i** Immunolabelling of internalised (green) and surface (red) GABA_A_Rs following 2 h treatments with diazepam (D; 1 μM; *n* *=* *2*; scale bar = 5 µm). **j** Diazepam (D; 1 μM)-dependent reduction of surface GABA_A_Rs is unaffected by inhibition of PP2A or PP1 with low (0.05 μM) or high (1 μM) dose of okadaic acid, respectively (OA; *n* *=* *6*), but **k** it is prevented by inhibition of calcineurin by cyclosporine A (CyA; 1 μM; *n* *=* *6*). **l** Diazepam (D; 1 μM) /isoguvacine (I; 5 μM)-dependent reduction in surface GABA_A_Rs in α_1_/β_2_/γ_2_^myc^-HEK293 cell line is prevented by inhibition of calcineurin with cyclosporine A (CyA; 1 μM; *n* *=* *3*). **m** Diazepam treatments (D; 1 μM; 2 h) cause GABA_A_R γ2 subunit dephosphorylation at Ser327 in cortical neurones and this is prevented by cyclosporine A (CyA; 1 μM; *n* *=* *2*). Immunoblotting was done using an anti-PSer327-γ2 or anti-γ2 primary antibody, followed by alkaline phosphatase-conjugated secondary antibody and a colour reaction. Quantification was done using ImageJ. **n** Diazepam (D; 1 μM)/isoguvacine (I; 5 μM)-dependent reduction in surface GABA_A_Rs in α_1_/β_2_/γ_2_^myc^-HEK293 cells is abolished by S327A mutation in the γ2 subunit. Changes in surface GABA_A_Rs were measured by cell surface ELISA using β_2/3_-specific antibody in cortical neurones or myc-antibody in α_1_/β_2_/γ_2_^myc^-HEK293 cells and presented in graphs as *mean* ± *s.e.m*., with *n* = *number of independent experiments*. Statistical analysis was done using ANOVA with Bonferonni post-hoc test; *p < *0.05*

In correlation with diazepam-dependent changes in postsynaptic GABA_A_Rs clusters observed in primary cerebrocortical neurones, a significant overall reduction in GABA_A_R surface expression was detected using a GABA_A_R β_2/3_-specific antibody in cell-surface ELISA experiments (Fig. 2). However, a decrease in cell surface levels was detected earlier than the observed disassembly of GABAergic synapses, reaching statistical significance within 1 h, and steady level after 24 h, in the continuous presence of diazepam (Fig. 2a, black line; *p < 0.05). In parallel ELISAs, in which the total levels of GABA_A_Rs were measured, a statistically significant decrease was detected after 72 h of continuous diazepam treatment (Fig. 2a, grey line; *p < 0.05). The effects of diazepam were not only time- but also dose-dependent, with the lowest effective concentration of 1 μM (Fig. 2b; *p < 0.05). A significant reduction in surface GABA_A_Rs was also observed in α1β2γ2^myc^-HEK293 cells treated with 1 μM diazepam in the presence of 5 μM isoguvacine (Fig. 2c, *p < 0.05). The observed decrease in surface GABA_A_Rs in neurones was abolished in the presence of Ro 15-1788 (Fig. 2d; *p < 0.05), again confirming that this process was initiated by direct diazepam binding to GABA_A_Rs. Moreover, this decrease in surface GABA_A_Rs was also abolished by picrotoxin, a GABA_A_R channel blocker, in neurones and in α1β2γ2^myc^-HEK293 cells (Fig. 2e, f, respectively; *p < 0.05), and by bicuculline (Fig. 2g; *p < 0.05), a competitive antagonist which blocked the binding of GABA to these receptors in neurones. Thus, the observed reduction in surface levels of GABA_A_Rs appears to be a consequence of prolonged diazepam-dependent stimulation of their activity evoked by endogenous GABA released from spontaneously active GABAergic neurones in culture (Fig. 1e) or by isoguvacine in α1β2γ2^myc^-HEK293 cells.

A time- and dose-dependent decrease in cell surface GABA_A_Rs was also observed in the continuous presence of specific agonists of these receptors at higher concentrations, muscimol (50 μM; Supplementary Figure 2; *p < 0.05) in primary neurons, or isoguvacine (50 μM; Fig. 2c; *p < 0.05) in α1β2γ2^myc^-HEK293 cells. Importantly, a statistically significant diazepam (1 μM)-dependent potentiation of muscimol effect at lower doses (1 or 5 μM muscimol; *p < 0.05), but not at higher doses (10 or 50 μM muscimol; Supplementary Figure 2a; *p* > 0.05) was observed, suggesting that, at higher agonist concentrations, GABA_A_Rs may have reached the maximal level of stimulation in these preparations. That GABA_A_R activation by muscimol was a necessary trigger for their downregulation from the cell surface was confirmed in neurones in the presence of bicuculline (Supplementary Figure 2c; *p < 0.05) or picrotoxin (Supplementary Figure 2d; *p < 0.05).

Crucially, the diazepam-dependent decrease in GABA_A_Rs surface levels in neurones was abolished in the presence of dynamin-inhibitory peptide (Fig. 2h; *p < 0.05), a peptide which is able to penetrate the plasma membrane and inhibit the endocytolic machinery of the cell [28]. This indicates that the underlying cause of reduction at the cell surface was indeed dynamin-dependent endocytosis of GABA_A_Rs rather than inhibition in protein synthesis or reduced insertion into the plasma membrane. Dynamin-inhibitory peptide also blocked a reduction in surface GABA_A_Rs caused by high doses of muscimol (Supplementary Figure 2e; *p < 0.05). Consistent with this was the loss of cell surface receptors (Fig. 2i, red) and a concomitant intracellular accumulation of endocytosed GABA_A_Rs (Fig. 2i, green) in the presence of diazepam (1 μM) or muscimol (50 μM; Supplementary Figure 2f), which were monitored using immunolabelling with the β_2/3_ antibody and confocal imaging in neurones. A small amount of internalised β_2/3_ subunits observed in neurones treated with vehicle control was likely due to constitutive internalisation of GABA_A_Rs [19]. Likewise, a reduction of surface GABA_A_Rs (Supplementary Figure 3b; red), with a concomitant intracellular accumulation of the endocytosed receptors (Supplementary Figure 3b; green), was also observed in α_1_β_2_γ_2_^myc^-HEK293 cells (Supplementary Figure 3a) treated with the submaximal doses of isoguvacine (5 μM), and this was further potentiated by the addition of diazepam (1 μM).

Dynamin-dependent endocytosis of GABA_A_Rs is known to be regulated by the activity of protein kinases and phosphatases which determine the state of phosphorylation of specific residues in the β and γ subunits of these receptors [29], such that dephosphorylation by protein phosphatase 1 or 2A [16], or calcineurin (Ca^2+^/calmodulin-dependent phosphatase 2B [30]), respectively, promotes their internalisation. To establish which of these phosphatases are involved in diazepam-triggered endocytosis of GABA_A_Rs, treatments of cultured neurones were carried out in the presence of either low (0.05 μM) or high (1 μM) doses of okadaic acid, to inhibit PP2A or PP2A/PP1, respectively, or in the presence of cyclosporine A (1 μM), to inhibit calcineurin, and surface GABA_A_Rs were monitored using cell surface ELISA. Although inhibition of PP2A alone, or both PP2A and PP1, had no effect (Fig. 2j), inhibition of calcineurin significantly attenuated GABA_A_R endocytosis triggered by diazepam in neurones (1 μM; Fig. 2k; *p < 0.05), or diazepam/isoguvacine (1 μM/5 μM; Fig. 2l, *p < 0.05) in α_1_β_2_γ_2_^myc^-HEK293 cells, suggesting that dephosphorylation of the γ_2_ subunit by calcineurin is a necessary step in this process. This was corroborated in immunoblotting experiments with P-Ser^327^-γ_2_-specific antibody, in which diazepam-dependent dephosphorylation of the γ_2_ subunit in neurones was abolished by cyclosporine A (Fig. 2m; *p < 0.05), and further supported by cell surface ELISA experiments in which mutated S327A γ2^myc^ subunit [31] was expressed in the α1β2-HEK293 cell line (Fig. 2n; *p < 0.05).

**Fig. 3:**
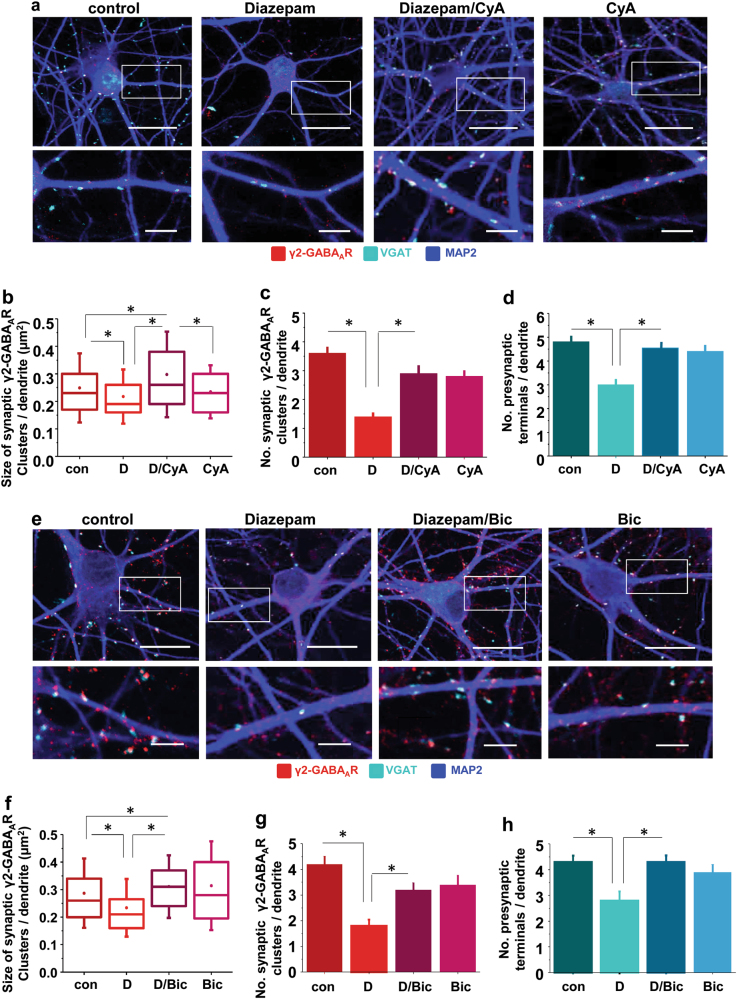
Diazepam-dependent loss of GABAergic synapses is prevented by inhibition of calcineurin or GABA_A_R activity. **a** Immunolabelling of γ2-containing clusters (red) and VGAT-positive presynaptic GABArgic terminals (cyan) along MAP2-positive dendrites (20 µm; blue) following 72 h treatment with control (DMSO) or diazepam (D; 1 μM), in the absence or presence of cyclosporine A (CyA; 1 μM), and corresponding graphs showing **b** size (median/line-IQRs; mean/dot ± s.d. whiskers; Mann–Whitney test, *p < *0.05*), and **c** number of synaptic γ_2_ clusters (mean ± s.e.m.; ANOVA/Bonferonni post-hoc test; *p < 0.05), from *n* = 71 control dendrites & *n* = *85*, *n* = 64, *n* = 73 dendrites of diazepam-, diazepam/cyclosporine A and cyclosporine A-treated cells, respectively, of a total of *n* *=* *19*, *n* *=* *19*, *n* *=* *18*, *n* *=* *19* neurons in each group collected from two independent experiments. **d** Decrease in number of GABAergic terminals (mean ± s.e.m.; ANOVA/Bonferonni post-hoc test; *p < 0.05) contacting *n* *=* *71* control-treated & *n* *=* *85*, *n* *=* *64*, *n* *=* *73* diazepam-, diazepam/cyclosporine A- or cyclosporine A-treated dendrites for 72 h, respectively, from a total of *n* *=* *19*, *n* *=* *19*, *n* *=* *18*, *n* *=* 19 neurons in each group, collected from two independent experiments. **e** Immunolabelling of γ2-containing clusters (red) and VGAT-positive presynaptic GABArgic terminals (cyan) along MAP2-positive dendrites (20 µm; blue) following 72 h treatment with control (DMSO) or diazepam (D; 1 μM), in the absence or presence of bicuculline (Bic; 50 μM), and corresponding graphs showing **f** size (median/line-IQRs; mean/dot ± s.d. whiskers; Mann–Whitney test, *p < 0.05), and **g** number of synaptic γ_2_ clusters (mean ± s.e.m.; ANOVA/Bonferonni post-hoc test; *p *<* *0.05*), from *n* *=* 60 control dendrites & *n* *=* 63, *n* *=* 61, *n* = 65 dendrites of diazepam-, diazepam/bicuculline and bicuculline-treated cells, respectively, of a total of *n* = 16, *n* = 19, *n* = 18, *n* = 20 neurons in each group collected from two independent experiments. **h** Decrease in number of GABAergic terminals (mean ± s.e.m.; ANOVA/Bonferonni post-hoc test; *p *<* *0.05*), contacting *n* = 60 control-treated & *n* = 63, *n* = 61, *n* *=* 65 diazepam-, diazepam/bicuculine- or bicuculline-treated dendrites for 72 h, respectively, from a total of *n* = 16, *n* = 19, *n* = 18, *n* = 20 neurons in each group, collected from two independent experiments. Scale bars = 20 μm (**a**, **e**-upper row) and = 5 μm (**a**, **h**-lower row)

The relationship between diazepam-dependent dephosphorylation and internalisation of GABA_A_Rs and subsequent destabilisation of GABAergic synapses was further investigated in experiments in which changes in synaptic elements, the size and number of postsynaptic γ_2_-GABA_A_R clusters and the number of colocalised presynaptic GABAergic terminals, were quantitatively assessed following prolonged diazepam treatments (72 h) in the absence or presence of cyclosporine A (Fig. 3a–d). The decrease in size (Fig. 3a, b; *p < 0.05) and number of postsynaptic γ_2_-GABA_A_R clusters (Fig. 3a, c; *p < 0.05), and in the number of colocalised presynaptic, VGAT-terminals (Fig. 3a, d; *p < 0.05) detected after 72 h treatment with diazepam, were effectively abolished in the presence of cyclosporine A, further supporting the central role of calcineurin in this process. Moreover, cyclosporine A and diazepam, when applied together, caused a significant increase in the size of GABA_A_R clusters in comparison with the control, diazepam or cyclosporine A alone (Fig. 3a, b; *p < 0.05).

Furthermore, a functional link between prolonged diazepam-dependent stimulation of GABA_A_Rs and disassembly of GABAergic synapses, was assessed in experiments in which structural elements of synapses were analysed in the presence of bicuculline (Fig. 3e–h). Bicuculline significantly attenuated the diazepam-dependent decrease in size (Fig. 3e, f; *p < 0.05) and number of postsynaptic γ_2_-GABA_A_R clusters (Fig. 3e, g; *p < 0.05), and in the number of colocalised presynaptic VGAT-terminals (Fig. 3e, h; *p < 0.05). Diazepam and bicuculline added together also caused a significant increase in the size of GABA_A_R clusters in comparison with control, diazepam or bicuculline treatments alone (Fig. 3e, f; *p < 0.05).

Collectively, these data evince a cascade of signalling events triggered by prolonged stimulation of synaptic GABA_A_Rs which, in turn, leads to a calcineurin-mediated dephosphorylation and endocytosis of these receptors, and a consequent disassembly of GABAergic synapses. As GABA_A_Rs are GABA-gated chloride/bicarbonate channels which are impermeable to Ca^2+^, while calcineurin activation requires an increase in cytoplasmic Ca^2+^, the question arises as to the nature of the signalling molecules that link the activities of these seemingly unrelated pathways.

### Diazepam triggers Ca^2+^ release from thapsigargin-sensitive intracellular stores by activating PLC

**Fig. 4:**
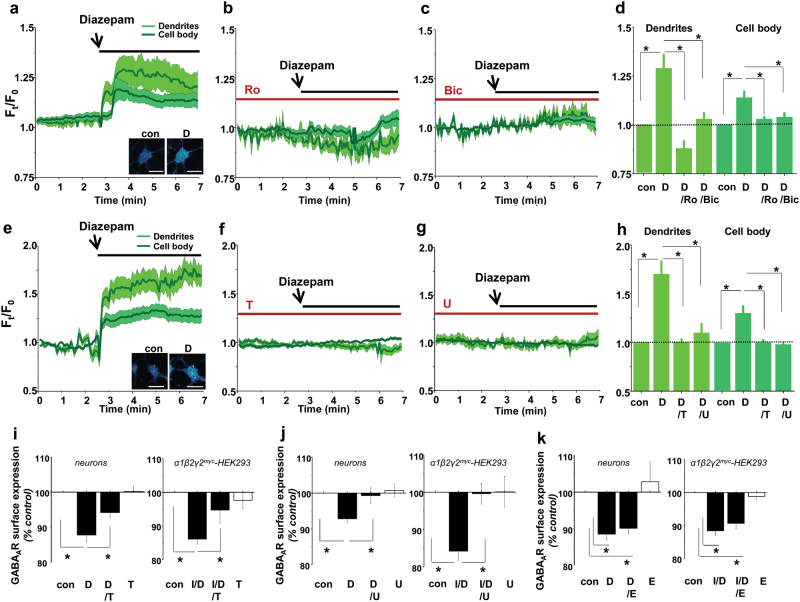
Diazepam triggers release of Ca^2+^ from the intracellular stores which is required for internalisation of GABA_A_Rs and prevented by Ro 15-1788, bicuculline, thapsigargin and U-73122. Time lapse imaging of intracellular Ca^2+^ in Fluo-4-labelled cortical neurones treated with diazepam (D; 1 μM) alone (**a**, inset: representative images before and after diazepam addition; scale bars = 20 μm), or in the presence of Ro15-1788 (Ro, 25 μM; **b**) or bicuculine (Bic, 50 μM; **c**), shown as a fluorescence ratio F_t_/F_0_, and **d** quantified at the peak of response to diazepam in dendrites and somas (**d**; mean ± s.e.m.; ANOVA/Bonferonni post-hoc test; *p *<* *0.05*; *n* *=* *5* neurones in each group from 2 independent experiments). **e** Diazepam (D; 1 μM)-dependent increase in intracellular Ca^2+^ (insert: representative images before and after diazepam addition; scale bars = 20 μm) is inhibited by thapsigargin (T; 2 μM; **f**) and U-73122 (U; 10 μM; **g**). **h** F_t_/F_0_ was quantified at the peak of response to diazepam in dendrites and somas of labelled cortical neurons (mean ± s.e.m.; ANOVA/Bonferonni post-hoc test; *p *<* 0.05; *n* = 4 neurones in each group from 2 independent experiments). **i–k** Diazepam (D; 1 μM)-dependent internalisation of GABA_A_Rs in neurones (left), and Diazepam (D; 1 μM)/Isoguvacine (I; 5 μM)-dependent internalisation of GABA_A_Rs in α_1_/β_2_/γ_2_^myc^-HEK293 cells (right) are prevented by thapsigargin (T; 2 μM; **i**) and U-73122 (U; 10 μM; **j**), but insensitive to EGTA (E; 1 mM; **k**). Changes in surface GABA_A_Rs were measured by cell surface ELISA using β_2/3_-specific antibody in neurones or myc-antibody in α_1_/β_2_/γ_2_^myc^-HEK293 cells and presented in graphs as mean ± s.e.m. Statistical analysis was done using ANOVA with Bonferonni post-hoc test; *p *<* *0.05* (*n* *=* *7* thapsigargin, *n* = 5 U-73122, *n* = 9 EGTA independent experiments)

To investigate the functional link between GABA_A_Rs and calcineurin, we monitored the intracellular Ca^2+^ signal using the Ca^2+^-indicator Fluo-4 in primary neurones by live cell imaging, which has revealed that application of diazepam (1 μM) evokes a prolonged increase in intracellular Ca^2+^in neuronal dendrites and cell bodies (Fig. 4a, d, e, h; *p < 0.05). Importantly, this increase was completely abolished when diazepam was applied in the presence of Ro 15-1788 (Fig. 4b, d; *p < 0.05) or bicuculline (Fig. 4c, d; *p < 0.05). Furthermore, the rise in intracellular Ca^2+^ was also abolished when the sarco/endoplasmic Ca^2+^ stores were depleted in the presence of thapsigargin, a non-competitive inhibitor of Ca^2+^ ATPase [32], prior to the addition of diazepam (Fig. 4f, h; *p < 0.05). These findings indicate a critical role of the intracellular Ca^2+^ stores in this process and are consistent with a metabotropic-type signalling classically mediated by phospholipase C (PLC) [33]. To characterise this process further, live imaging of Fluo-4 labelled neurones was carried out in the presence of a general PLC inhibitor U73122 (10 µM), which effectively abolished the increase in intracellular Ca^2+^ in response to diazepam-dependent activation of GABA_A_Rs (Fig. 4g, h; *p < 0.05). In agreement with this, diazepam-dependent down-regulation of surface GABA_A_Rs in neurones and in α_1_β_2_γ_2_^myc^-HEK293 cells was also attenuated by thapsigargin (Fig. 4i, left and right, respectively; *p < 0.05) and U73122 (Fig. 4j; left and right, respectively; *p < 0.05), but not by extracellular chelation of Ca^2+^ in the presence of EGTA (Fig. 4k; left and right, respectively; *p < 0.05).

**Fig. 5:**
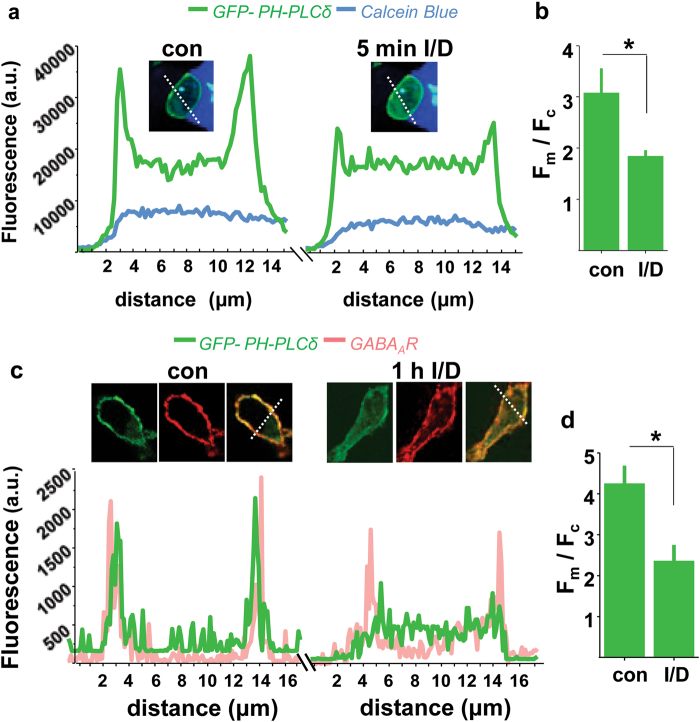
Diazepam/Isoguvacine-dependent translocation of GFP-PH_PLCδ1_ from the cell membrane to the cytoplasm in α_1_/β_2_/γ_2_-HEK293 cells. **a** Live imaging of a Calcein blue-labelled cell (inset) showing changes in GFP-PH_PLCδ1_ fluorescence intensity profile (green) prior to (left) and 5 min after the addition of Diazepam (D; 1 μM)/Isoguvacine (I; 5 μM) (right). **b** Quantification of fluorescence F(membrane)/F(cytoplasm) ratio of GFP-PH_PLCδ1_ (green; mean ± s.e.m.; Student *t*-test; *p < 0.05; *n* *=* 10 cells from 2 independent experiments). **c** Imaging of surface GABA_A_R-β_2_-subunit in fixed HEK293 cells (red) expressing GFP-PH_PLCδ1_ (green) in control (DMSO, left) and Diazepam (D; 1 μM)/Isoguvacine (I; 5 μM) treated samples for 1 h (right), showing superimposed fluorescence intensity profiles across the selected cells. **d** Quantification of fluorescence F(membrane)/F(cytoplasm) ratio of GFP-PH_PLCδ1_ (green; mean ± s.e.m*.*; Student *t*-test; *p < 0.05; *n* = 10 cells from 2 independent experiments)

To monitor the activation of PLC by diazepam/isoguvacine, an indirect method was employed in which GFP-tagged PH domain of PLCδ (GFP-PH_PLCδ_ [23]) was expressed in α_1_β_2_γ_2_-HEK293 cell line [18]. In confocal live cell imaging experiments, due to activation of endogenous PLC and depletion of PIP_2_ in response to diazepam/isoguvacine, the GFP-PH_PLCδ_, initially predominantly plasma membrane bound (F_m_), showed partial translocation to the cytoplasm (F_c_) within 5 min (green traces; Fig. 5a), resulting in a significant decrease in F_m_/F_c_ ratio in comparison with controls (Fig. 5b; *p < 0.05). That this activation was long-lasting was shown by fluorescent imaging of GFP-PH_PLCδ_ in α_1_β_2_γ_2_-HEK293 cells (green traces; Fig. 5c), which were fixed after 1 h in continuous presence of diazepam/isoguvacine and immunolabelled with GABA_A_R-β_2_-specific antibody at the cell surface (pink traces; Fig. 5c), thus yielded not only a significant decrease in F_m_/F_c_ ratio (Fig. 5d; *p < 0.05), but also a decrease in surface GABA_A_Rs.

Collectively, these data indicate that sustained diazepam-dependent activation of GABA_A_Rs in neurones, beyond its ionotropic effects, also has a long-lasting metabotropic effect mediated by a PLC/Ca^2+^/calcineurin signalling cascade which facilitates receptor internalisation from the cell surface.

### Regulation of GABA_A_R interaction with PLCδ or PRIP1 by diazepam

**Fig. 6:**
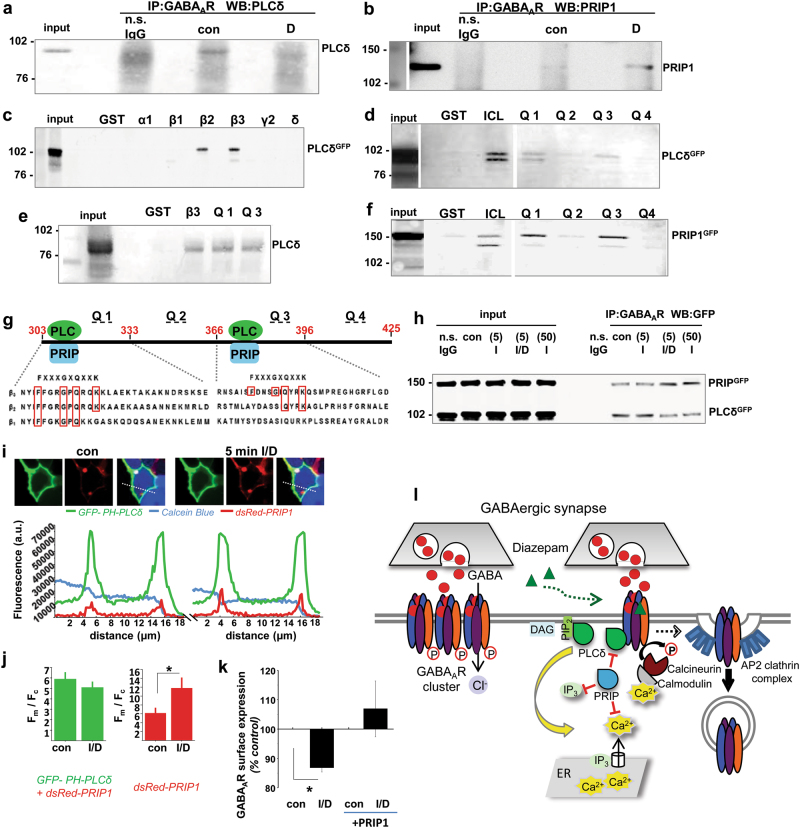
Diazepam triggers dissociation of PLCδ from GABA_A_Rs in situ leading to activation of PLCδ/Ca^2+^/calcineurin signalling pathway, which is negatively regulated by PRIP1. **a** Immunoprecipitates of GABA_A_Rs from control and diazepam (D; 1 μM)-treated cortical neurones were probed with PLCδ- (*n* *=* 3) or **b** PRIP1-  (*n* = 4) specific antibody. **c**–**e** PLCδ-GFP binds directly to the intracellular loop of the GABA_A_R β_2_ and β_3_ subunits Q1 (303-366 aa) and Q3 (366-396 aa) regions in GST pull-down assays (*n* = 3). **f** PRIP1-GFP binds directly to the β_3_ subunit Q1 and Q3 loop regions in the GST pull-down assays (*n* = 3). **g** Predicted PLCδ- and PRIP1- binding sites in the Q1 and Q3 regions of the β_2_ and β_3_ subunits. **h** Immunoprecipitates of GABA_A_Rs from control (DMSO) or Isoguvacine (I; 5 μM)-, Diazepam (D; 1 μM)/Isoguvacine (I; 5 μM)- or Isoguvacine (I; 50 μM)-treated α_1_β_2_γ_2_-GABA_A_R HEK293 cells expressing both GFP-PLCδ and GFP-PRIP1 were probed with the GFP-specific antibody (*n* = 2). (**i**) Overexpression of PRIP1 inhibits partial translocation of GFP-PH_PLCδ1_ in response to Diazepam (D; 1 μM)/Isoguvacine (I; 5 μM) in α_1_β_2_γ_2_-HEK293 cells. Live imaging of a Calcein blue-labelled cell (blue) expressing GFP-PH_PLCδ1_ (green) and dsRed-PRIP1 (*red*; top panels) and superimposed fluorescence intensity profiles prior to (left) and 5 min after the addition of Diazepam (D; 1 μM)/Isoguvacine (I; 5 μM) (right). **j** Quantification of fluorescence F(membrane)/F(cytoplasm) ratio of GFP-PH_PLCδ1_ (green; left) and dsRed-PRIP1 (red; right), both shown as mean ± s.e.m. (Student *t*-test; *p < 0.05; *n* = *10* cells from 2 independent experiments). **k** Overexpression of PRIP1 inhibits Diazepam (D; 1 μM)/Isoguvacine (I; 5 μM)-dependent internalisation of GABA_A_Rs. Changes in surface GABA_A_Rs were measured by cell surface ELISA with anti-myc-specific antibody labelling the γ2 subunit, and presented as mean ± s.e.m. (*n* = 4). Statistical analysis was done using ANOVA with Bonferonni post-hoc test; *p *<* *0.05*; *n* = number of independent experiments. **l** Schematic diagram of the GABA_A_R/PLCδ/Ca^2+^/calcineurin feed-back mechanism underlying diazepam-dependent downregulation of GABA_A_Rs. According to this model, sustained activation of synaptic GABA_A_Rs by diazepam triggers a metabotropic, PLCδ/Ca^2+^/calcineurin signalling pathway which leads to receptor dephosphorylation by calcineurin, initiation of dynamin-dependent endocytosis resulting in a decrease in the size and number of postsynaptic GABA_A_R clusters, and disassembly of inhibitory synapses. This mechanism is ‘switched off’ when PRIP1, PLCδ-related but catalytically inactive protein, outcompetes the PLCδ in binding to GABA_A_Rs, thereby preventing the activation of PLCδ and downstream Ca^2+^/calcineurin-dependent internalisation of these receptors

Diazepam/isogivacine-dependent activation of PLC and its requirement in diazepam-dependent mobilisation of intracellular Ca^2+^ suggests that GABA_A_Rs may be in association with some of the 23 known isoforms of PLC [33], via an interaction that might directly impinge on the activity of these enzymes. To test this hypothesis, the PLCδ isoform was initially investigated given its structural similarity to PRIP1 (PLC-related but catalytically inactive protein 1[34]), which has been previously shown to directly associate with GABA_A_Rs [35]. Coimmunoprecipitation experiments from control and diazepam-treated neurones demonstrated that PLCδ binds to GABA_A_Rs in controls, but disassociates from them when diazepam is applied (Fig. 6a), while PRIP1 shows the opposite translocation (Fig. 6b). To investigate these interactions further, in vitro binding assays were carried out in which purified GST-fusion proteins incorporating the intracellular TM3-4 loop of different GABA_A_R subunits were incubated with lysates of PLCδ-GFP expressing HEK293 cells, revealing that PLCδ binds to the β2 and β3 subunits of GABA_A_Rs specifically (Fig. 5c). Using truncation mutants of the β3 subunit TM3-4 loop, two distinct binding regions, between 303–333 (Q1) and 366–396 (Q3) residues, were identified (Fig. 5d) and, in subsequent experiments, demonstrated to mediate the direct binding of PLCδ (Fig. 5e). Interestingly, PRIP1-GFP was also found to interact with the same Q1 and Q3 regions of the GABA_A_R β3 subunit as PLCδ (Fig. 5f), suggesting that, in situ, these two proteins may be in competition for their binding to GABA_A_Rs. Based on the sequence similarity in the Q1 and Q3 regions between the β_2_ and β_3_ subunits, a potential binding sequence for both PLCδ and PRIP1 was identified (FXXXGXQXXK; Fig. 5g).

Dissociation of PLCδ from GABA_A_Rs upon their activation by diazepam/isoguvacine and the concurrent increase in PRIP1 binding were confirmed by coimmunoprecipitation from lysates of α1β2γ2-HEK293 cells transfected with GFP-PLCδ and GFP-PRIP1 cDNA (Fig. 5h), further supporting the observation that the binding of these proteins is regulated by the level of GABA_A_R activation, but in a mutually exclusive manner.

Altogether, the data suggest that a switch in association between GABA_A_Rs and catalytically-active PLCδ versus catalytically inactive PRIP1, may be a critical regulatory step in the signalling pathway that leads to diazepam-dependent internalisation of GABA_A_Rs. To test this hypothesis, the intracellular localisation of GFP-PH_PLCδ_ and dsRed-PRIP1 was monitored simultaneously by live cell imaging in transfected α1β2γ2-HEK293 cells before and 5 min after the bath application of diazepam/isoguvacine (Fig. 6i). GFP-PH_PLCδ_ showed no apparent translocation from the membrane to the cytoplasm (green traces, Fig. 6i) and no change in F_m_/F_c_ ratio (Fig. 6j, left) when dsRed-PRIP1 was also expressed, suggesting that activation of endogenous PLCδ in response to diazepam/isoguvacine was blocked. In contrast, dsRed-PRIP1 showed further accumulation in the plasma membrane (red traces, Fig. 6i) resulting in an increase in F_m_/F_c_ ratio (Fig. 6j, right). Moreover, a decrease in surface GABA_A_Rs in response to diazepam/isoguvacine in α_1_β_2_γ_2_^myc^-HEK293 cells detected by cell surface ELISA, was completely abolished by overexpression of PRIP1 (Fig. 5k), suggesting that PRIP1 may serve as an inhibitor of this signalling pathway, thereby preventing the process of GABA_A_Rs internalisation and alleviating the consequent loss of inhibitory GABAergic synapses (Fig. 5l).

## Discussion

The prevalence of stress-related psychiatric disorders, particularly anxiety mixed with depression, panic attacks or insomnia, leads to an estimated 12 million prescriptions of benzodiazepines every year in the UK (UK Addiction Treatment Centres). However, our current understanding of the long-term effects of benzodiazepines on cellular and molecular processes in the brain remains limited.

In this study we have revealed that prolonged exposure of neurons to diazepam activates a novel Ca^2+^ signalling cascade downstream of GABA_A_Rs, which in a negative feedback fashion, leads to a gradual removal of these receptors from the postsynaptic membrane and disassembly of inhibitory synapses, thus rendering the system unresponsive to any further diazepam treatments. Although studied in vitro, these processes are closely correlated in time to in vivo downregulation of GABA_A_Rs and the onset of tolerance to benzodiazepines in rodents [36]. These processes are however in sharp contrast with the initial diazepam-dependent facilitation of GABA_A_R channel gating activity [25], increased mobilisation of GABA_A_Rs to synapses [37, 38], and enhanced inhibitory synaptic transmission [39], possibly representing a form of neuronal adaptation in order to maintain a critical balance between the excitation and inhibition in the brain.

Our experiments demonstrate that sustained activation of GABA_A_Rs by diazepam, in the presence of ambient GABA (Fig. 1e [39]), is a key trigger of this signalling cascade which involves PLCδ activation, mobilisation of intracellular Ca^2+^ and activation of calcineurin. A key role of calcineurin in diazepam-dependent endocytosis of GABA_A_Rs is in agreement with the previously reported effects on GABA_A_R migration out of the synaptic contacts [31, 40, 41], and internalisation from the cell surface [42]. Subsequent loss of inhibitory synapses indicates that depletion of postynaptic GABA_A_Rs destabilises synaptic contacts, an observation consistent with their activity-dependent regulation reported previously [43], and also with a direct structural role of GABA_A_Rs in the formation of these synapses [17, 44, 45]. As changes in cell surface GABA_A_Rs are known to precede changes in the postsynaptic gephyrin scaffold [46], our findings are also in agreement with the previously observed diazepam-dependent reduction in the size of gephyrin clusters [47]. At later time points of incubation with diazepam, an overall reduction in GABA_A_R expression is likely to occur due to induced proteolysis of internalised receptors, as previously reported for the prolonged flurazepam treatments [48], or due to a decrease in the rate of transcription and/or translation of GABA_A_R subunits, as shown in vivo by monitoring the mRNA levels [10, 11, 15, 49].

Another important step in this cascade is diazepam-dependent rise in intracellular Ca^2+^ originating from the thapsigargin-sensitive intracellular stores and mediated by the activation of PLCδ, which is consistent with a metabotropic-type signalling by GABA_A_Rs in mature neurones operating in addition to their ‘canonical’ ionotropic signalling as chloride/bicarbonate channels. In contrast to immature neurones, where GABA_A_R activation generally leads to depolarisation and influx of Ca^2+^ through voltage-gated Ca^2+^ channels [50, 51], this signalling pathway is insensitive to the extracellular chelation of Ca^2+^ with EGTA. Furthermore, the requirement for PLC activity in this process is in agreement with the biochemical evidence for a direct association between GABA_A_R β subunits and PLCδ, which was also detected in recent proteomic analyses of GABA_A_R binding proteins [52, 53]. Although PLCδ may not be the only isoform of PLC able to interact with GABA_A_Rs, its extensive structural similarity with PRIP1, a well characterised GABA_A_R receptor interacting protein [54], is of significance. Interestingly, our experiments demonstrate that PLCδ disassociates from GABA_A_Rs with diazepam treatments, suggesting that this displacement may be required for its activation, particularly if the PLCδ binding to GABA_A_Rs resembles its binding to calmodulin via an auto-inhibitory region which renders it inactive [55]. Exactly how dissociation of PLCδ from GABA_A_Rs occurs remains to be elucidated, although we hypothesise that this could be caused by a conformational change in the receptor upon activation, or by accumulation of negatively charged chloride and depletion of bicarbonate, possibly changing the pH or osmolality [56] in the vicinity of the receptor. At the same time, however, these conditions facilitate the binding of PRIP1 to GABA_A_Rs, which, together with identified common binding sites in the β subunits, suggests that the two proteins may be in competition with each other. The interplay between PLCδ and PRIP1 in binding to GABA_A_Rs may represent an important on/off switching mechanism regulating this signalling pathway and downstream endocytosis of GABA_A_Rs. This is because PRIP proteins are not only catalytically inactive variants of PLCδ unable to generate IP_3_ and DAG [34], but they also inhibit PLC signalling due to high affinity binding of IP_3_, thereby sequestering it away from the IP_3_ receptor on the intracellular Ca^2+^ stores [57]. That overexpression of PRIP1 inhibited diazepam-dependent activation of PLCδ and diazepam-dependent down-regulation of surface GABA_A_Rs in heterologous systems, suggests that this protein can act as an inhibitor by outcompeting the PLCδ binding to GABA_A_Rs. In neurones, PRIP1 is likely to be a temporary block of this pathway, due to its removal together with GABA_A_Rs undergoing endocytosis [58]. This is in agreement with the effects of PRIP gene deletion in mice, which show increased intracellular Ca^2+^ and calcineurin activity [57], decreased levels of synaptic γ_2_-containing GABA_A_Rs receptors, reduced sensitivity to diazepam and increased anxiety-like behaviour [59]. We therefore hypothesise that, when the ‘off’ switch (PRIP) is no longer present, PLCδ recruitment to GABA_A_Rs allows continuous activation of this signalling pathway and depletion of synaptic GABA_A_ receptors, leading to their structural and functional deficits in inhibitory synapses. This diazepam-induced breakdown of inhibitory GABAergic synapses not only correlates well in time with the development of tolerance but also provides a likely explanation for the severe withdrawal symptoms, increased anxiety and even seizures, observed in animal models and patients following sudden termination of chronic benzodiazepine treatment [60], possibly due to uncontrolled excitatory drive in the absence of functional inhibition.

This signalling mechanism offers a new spectrum of possible molecular interventions that could be tailored towards extending the initial highly beneficial clinical outcomes of benzodiazepines, while preventing the subsequent disruption of GABAergic synapses and development of pharmacological and behavioural tolerance to these widely prescribed drugs.

## Electronic supplementary material

Supplementary Figure 1

Supplementary Figure 2

Supplementary Figure 3

Supplementary Figure 4

Supplementary Table 1

Supplementary Table 2

Supplementary Table 3

Supplemental Materials File #1
