# Supplementary figures and images for: Diazepam-induced loss of inhibitory synapses mediated by PLCδ/ Ca2+/calcineurin signalling downstream of GABAA receptors
Source: Mol Psychiatry. 2018 Jun 14;23(9):1851–67. doi: 10.1038/s41380-018-0100-y (PMC6232101; doi:10.1038/s41380-018-0100-y)

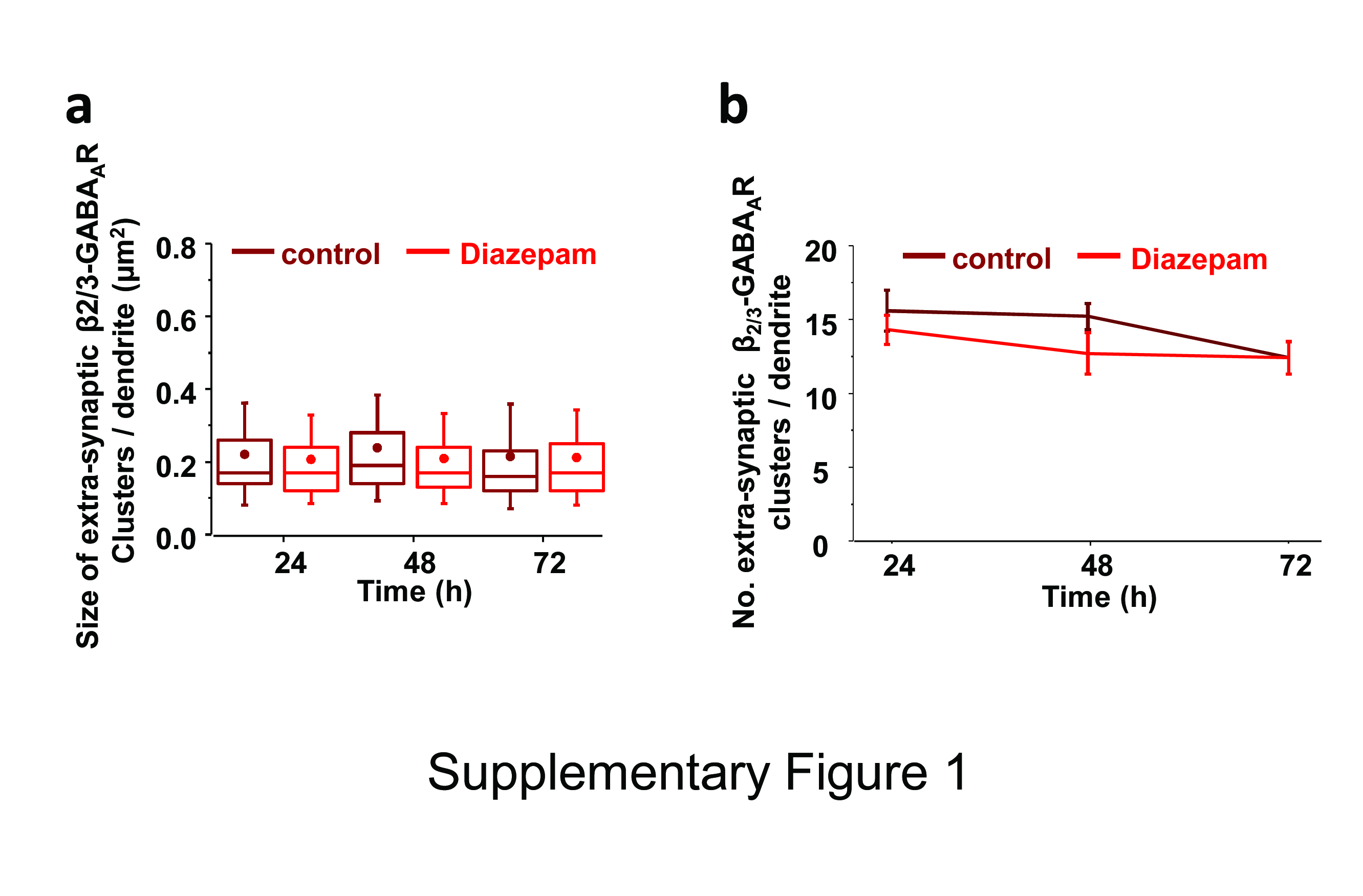

Supplement: Supplementary file 1 — Supplementary Figure 1 [file 41380_2018_100_MOESM1_ESM.tif]

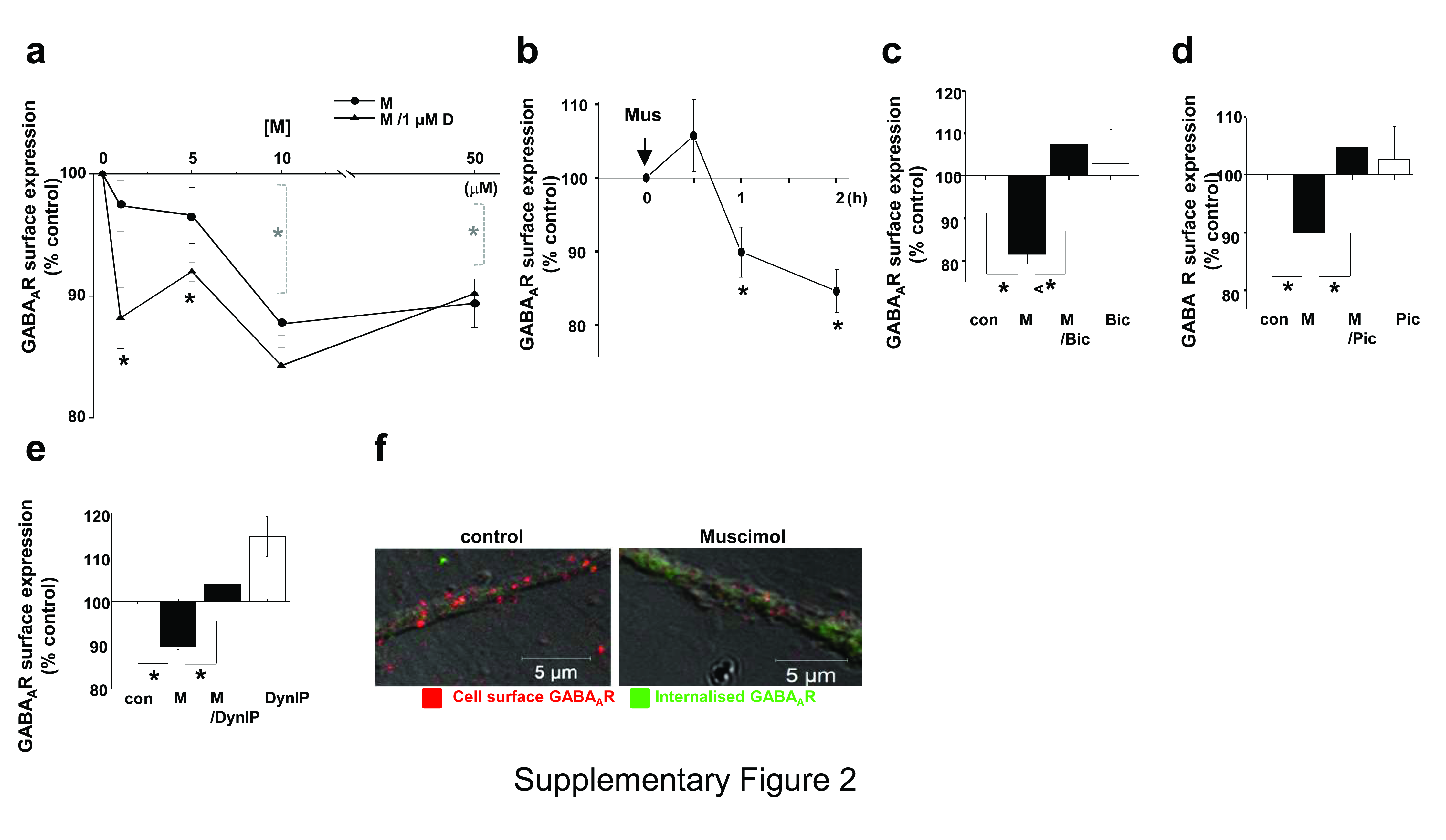

Supplement: Supplementary file 2 — Supplementary Figure 2 [file 41380_2018_100_MOESM2_ESM.tif]

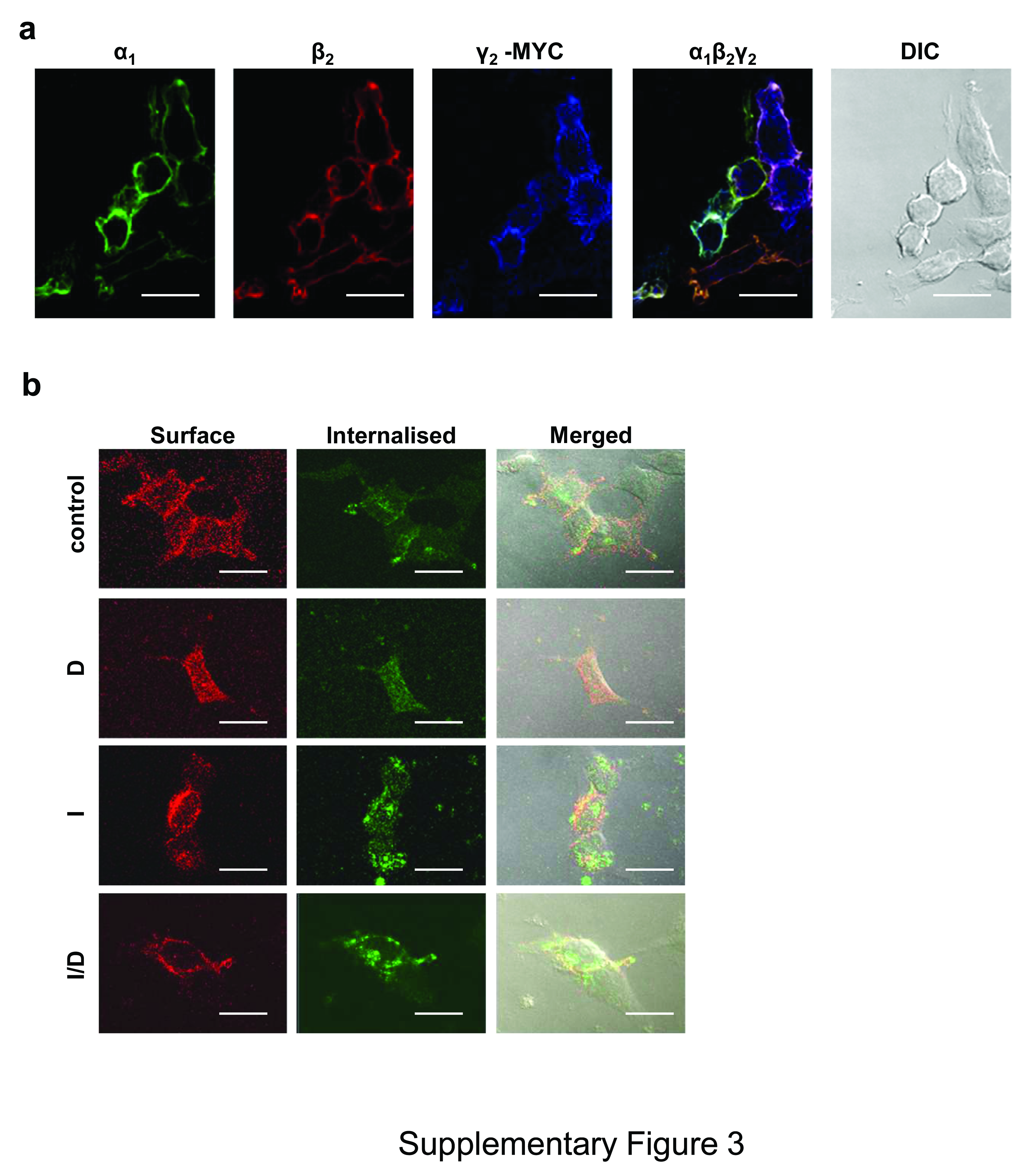

Supplement: Supplementary file 3 — Supplementary Figure 3 [file 41380_2018_100_MOESM3_ESM.tif]

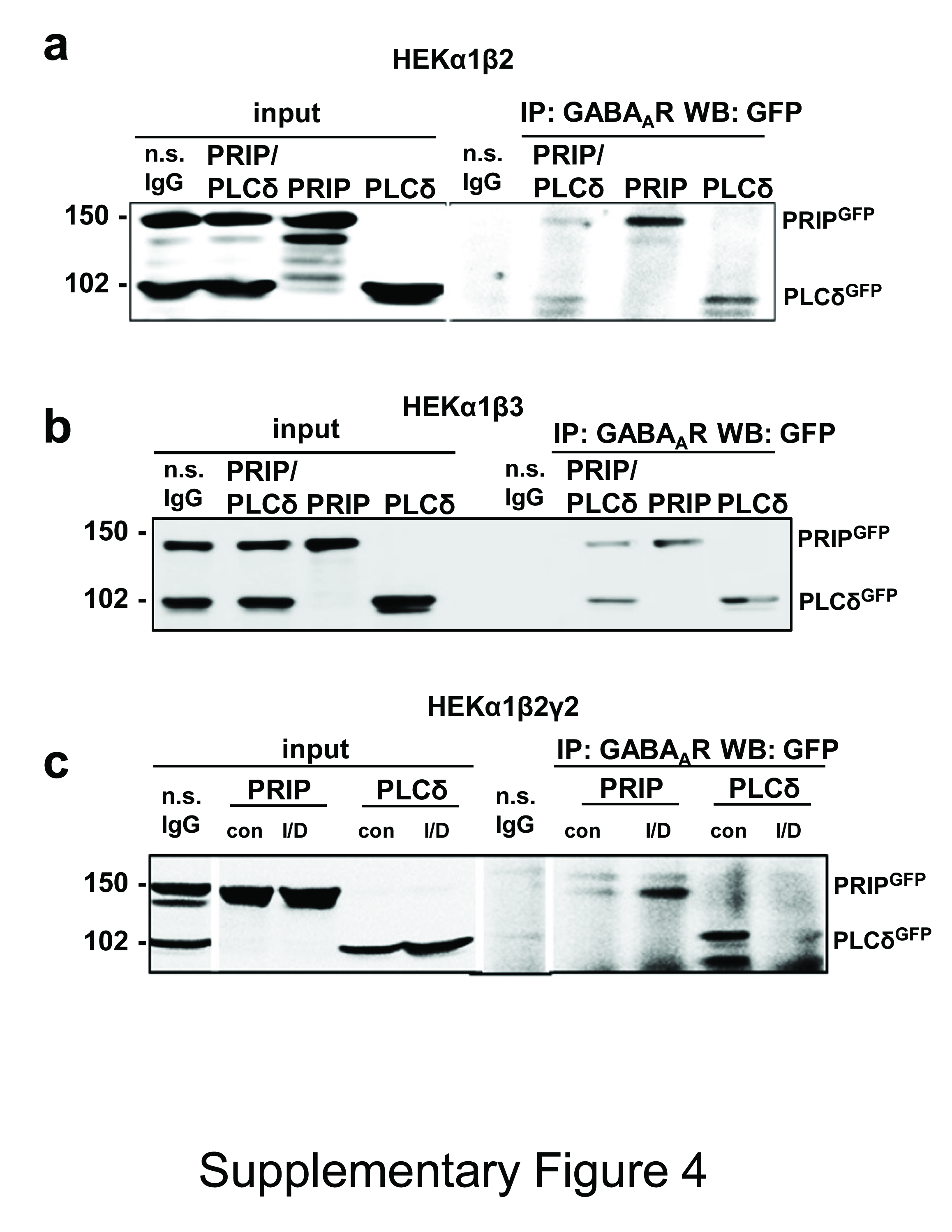

Supplement: Supplementary file 4 — Supplementary Figure 4 [file 41380_2018_100_MOESM4_ESM.tif]

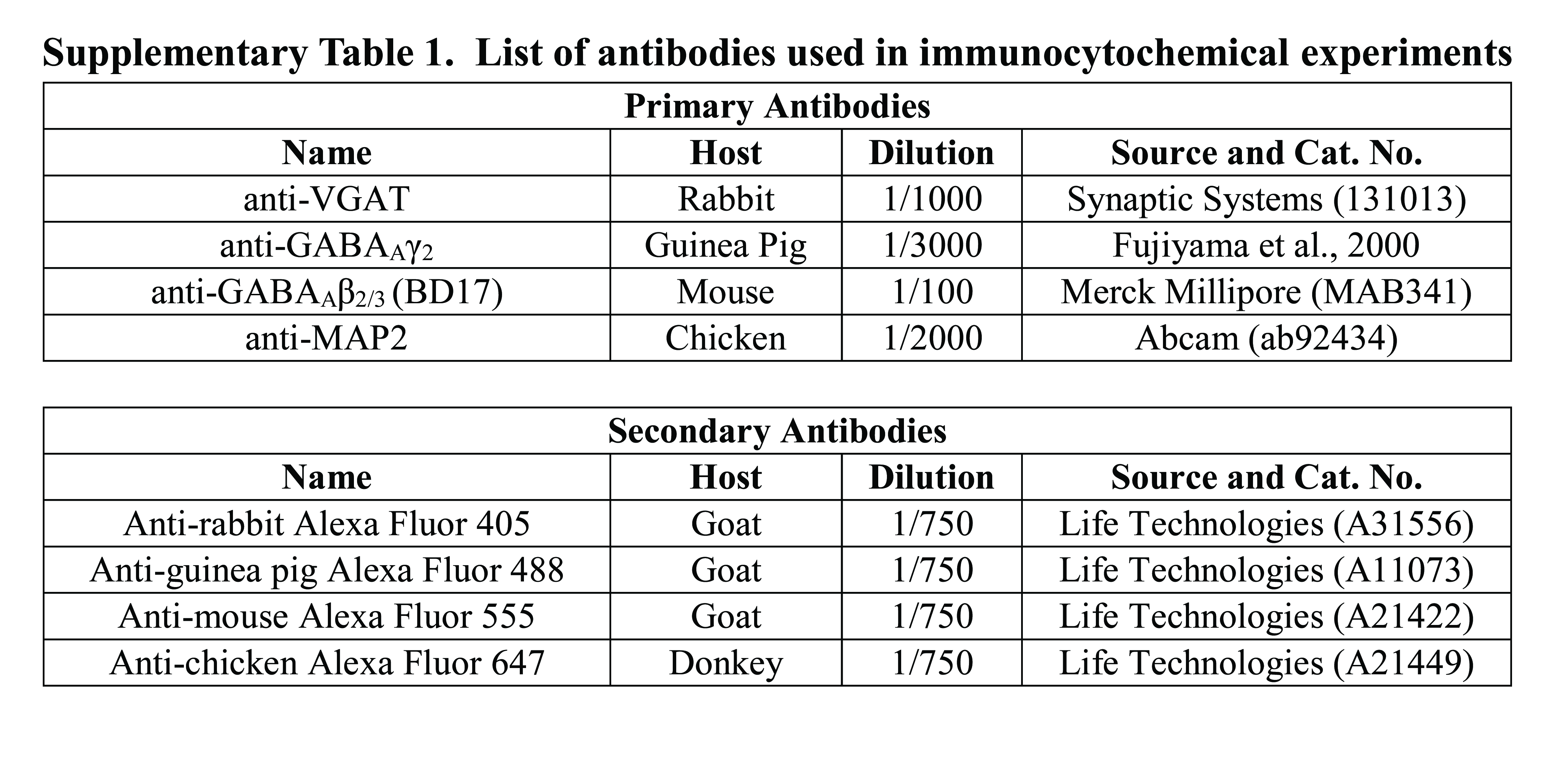

Supplement: Supplementary file 5 — Supplementary Table 1 [file 41380_2018_100_MOESM5_ESM.tif]

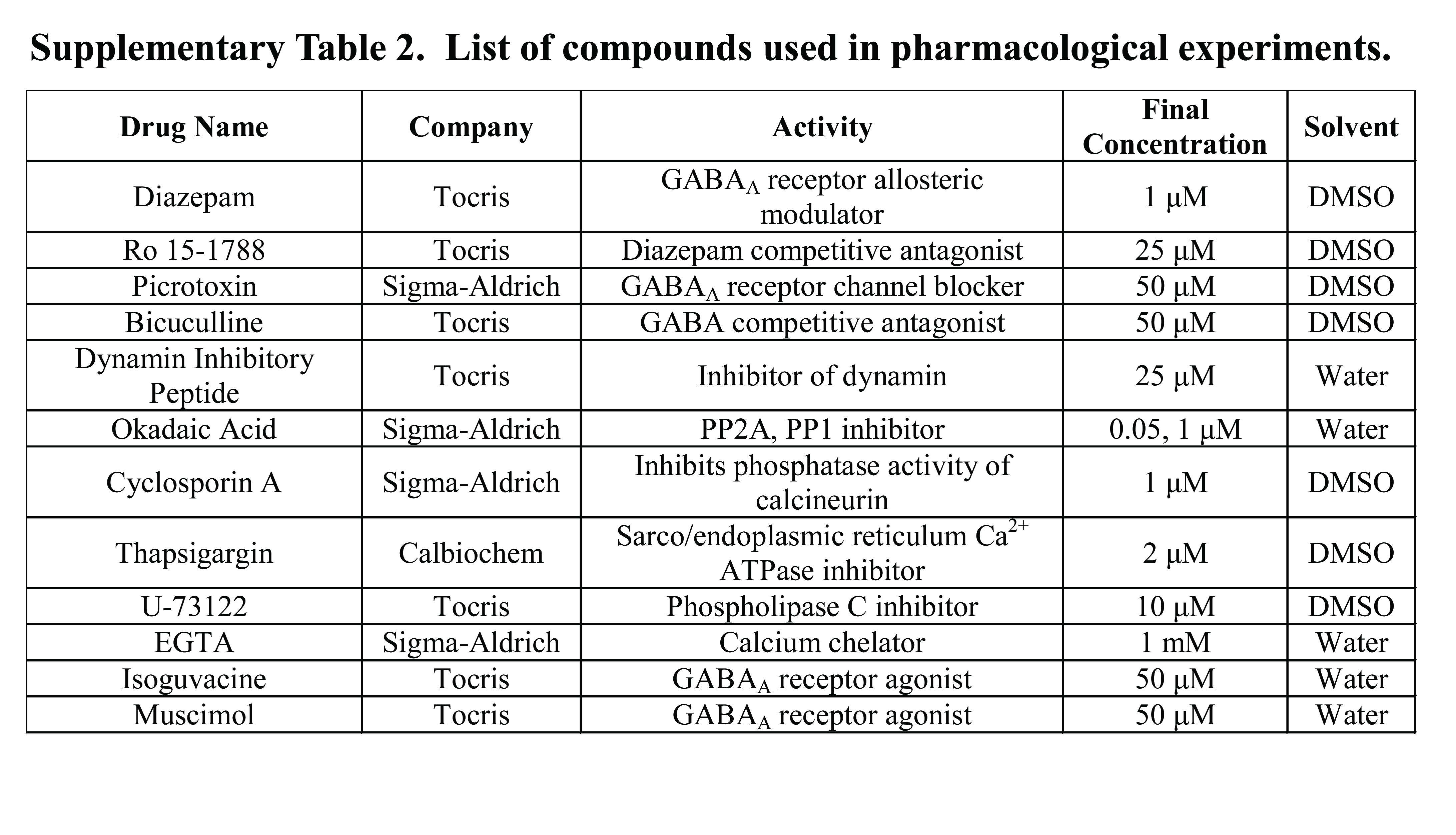

Supplement: Supplementary file 6 — Supplementary Table 2 [file 41380_2018_100_MOESM6_ESM.tif]

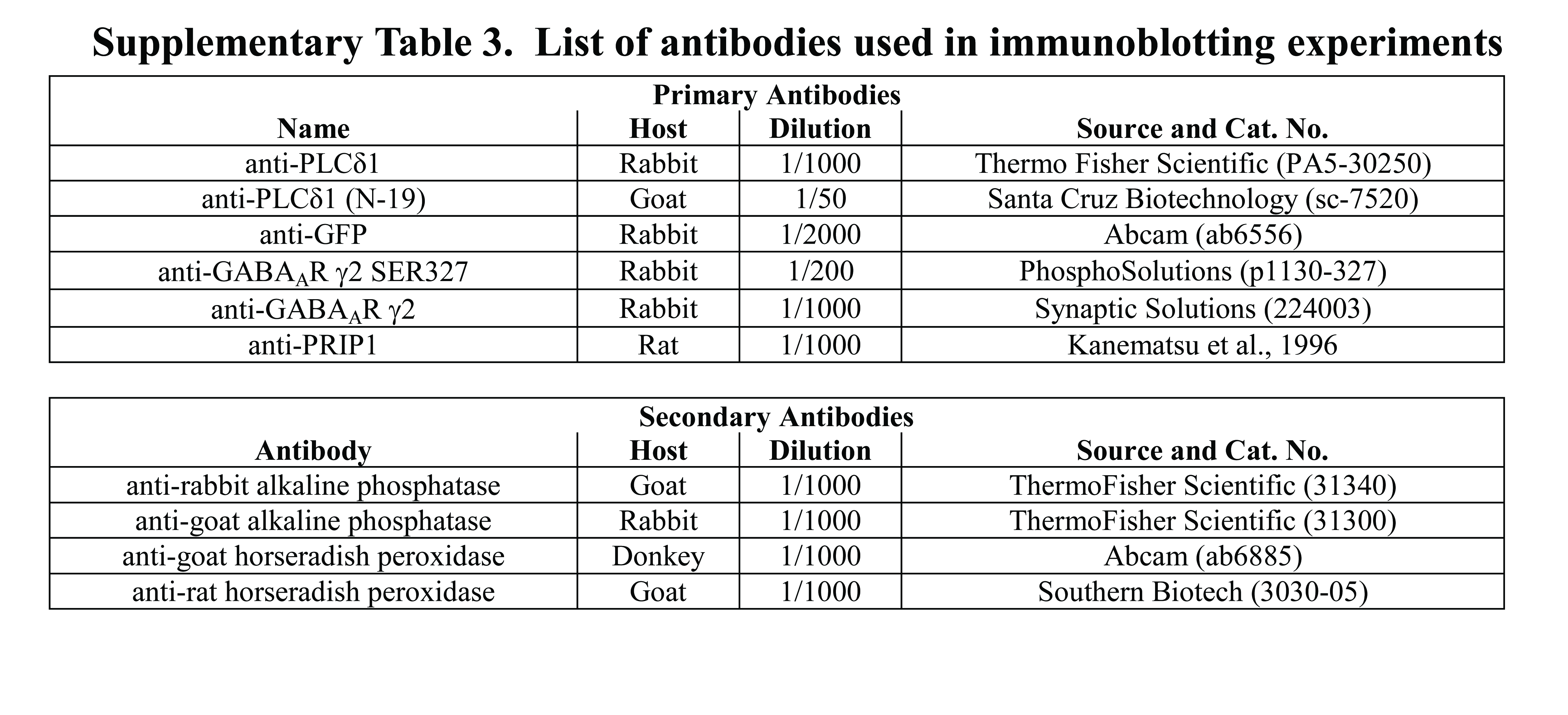

Supplement: Supplementary file 7 — Supplementary Table 3 [file 41380_2018_100_MOESM7_ESM.tif]
