# Supplementary material for: Diazepam-induced loss of inhibitory synapses mediated by PLCδ/ Ca2+/calcineurin signalling downstream of GABAA receptors
Source: Mol Psychiatry. 2018 Jun 14;23(9):1851–67. doi: 10.1038/s41380-018-0100-y (PMC6232101; doi:10.1038/s41380-018-0100-y)
Supplement: Supplementary file 8 — Supplemental Materials File #1 [file 41380_2018_100_MOESM8_ESM.docx]

Supplementary Figure 1.

The size and number of extrasynaptic GABA_A_R clusters are unaffected by diazepam treatments. (**a, b**) Graphs showing no change over time in (**a**) size (*median/line-IQRs; mean/dot±s.d. whiskers; Mann Whitney test, *p<0.05*) or (**b**) number (*mean±s.e.m.; ANOVA/Bonferonni post-hoc test; *p < 0.05*) of extrasynaptic β_2/3_ clusters, measured along the primary dendrites (*n=59*, *n=70* and *n=53* dendrites of control & *n=70*, *n=67*, and *n=44* dendrites of diazepam-treated cells) over 24 h, 48 h and 72 h, respectively, from a total of *n*=17, *n=*18 and *n=*15 control and *n*=17, *n=*17 and *n=*17 diazepam-treated neurons in each group, collected from two independent experiments.

Supplementary Figure 2.

Dose- and time-dependent internalisation of GABA_A_Rs in response to muscimol. (**a**) Muscimol (M) -dependent decrease in surface levels of GABA_A_Rs was potentiated by diazepam (1 μM) at submaximal doses (1 & 5 µM; *n=6; *p<0.05, black),* but not at maximal doses (10 & 50 µM*)* which alone produced a significant downregulation (*n=6; *p<0.05; gray) .* (**b**) Time-course of muscimol (M; 50 µM)-dependent decrease in surface GABA_A_Rs (*n=4)*. (**c-e**) Muscimol (M; 50 µM)-dependent decrease in surface GABA_A_Rs was inhibited by (**c**) bicuculline (Bic, 50 µM; *n=4*), (**d**) picrotoxin (Pic, 50 µM; *n=4*)*,* and (**e**) dynamin-inhibitory peptide (DynIP, 25 µM; *n=8*), respectively, following 2 h treatments. (**f**) Immunolabelling of internalised (*green*) and surface (*red*) GABA_A_Rs following 2 h muscimol (50 µM) treatments (*n=2;* scale bar=5 µm). Changes in surface GABA_A_Rs were measured by cell surface ELISA using β_2/3_-specific antibody and presented in graphs as *mean±s.e.m*. Statistical analysis was done using ANOVA with Bonferonni post-hoc test; **p<0.05; n=number of independent experiments.*

Supplementary Figure 3.

(**a**) Immunolabelling of surface GABA_A_Rs with subunit-specific antibodies in α_1_/β_2_/γ_2_^myc^-HEK293 cells (*n=2*, scale bar=20 μm). (**b**) Immunolabelling of internalised (*green*) and surface (*red*) GABA_A_Rs following 2 h treatment with control (DMSO) or Diazepam (D; 1 µM), Isoguvacine (I; 5 µM) or Diazepam/Isoguvacine, superimposed on DIC images (merged; *n=2;* scale bar=20 µm).

Supplementary Figure 4.

Competitive binding of PLCδ and PRIP1 to GABA_A_Rs is regulated by diazepam/isoguvacine in α_1_/β_2_/γ_2_-HEK293 cells. (**a-b**) Immunoprecipitates of GABA_A_Rs from α_1_β_2_- (**a**) or α_1_β_3_- (**b**) α_1_/β_2_/γ_2_-HEK293 cells expressing GFP-PLCδ and GFP-PRIP1 together or individually, probed with the GFP-specific antibody (*n=2).* (**c**) Immunoprecipitates of GABA_A_Rs from control (DMSO) or Diazepam (D; 1 μM)/Isoguvacine (I; 5 μM)-treated α_1_β_2_γ_2_-HEK293 cells, expressing either GFP-PLCδ or GFP-PRIP1, probed with the GFP-specific antibody (*n=2).*
